# Network inoculation: Heteroclinics and phase transitions in an epidemic model

**DOI:** 10.1063/1.4961249

**Published:** 2016-08-22

**Authors:** Hui Yang, Tim Rogers, Thilo Gross

**Affiliations:** 1 Web Sciences Center, University of Electronic Science and Technology of China, Chengdu 610054, China; 2 Big Data Research Center, University of Electronic Science and Technology of China, Chengdu 610054, China; 3 Department of Engineering Mathematics, University of Bristol, Bristol BS8 1UB, United Kingdom; 4 Centre for Networks and Collective Behaviour, Department of Mathematical Sciences, University of Bath, Claverton Down, BA2 7AY Bath, United Kingdom

## Abstract

In epidemiological modelling, dynamics on networks, and, in particular, adaptive and heterogeneous networks have recently received much interest. Here, we present a detailed analysis of a previously proposed model that combines heterogeneity in the individuals with adaptive rewiring of the network structure in response to a disease. We show that in this model, qualitative changes in the dynamics occur in two phase transitions. In a macroscopic description, one of these corresponds to a local bifurcation, whereas the other one corresponds to a non-local heteroclinic bifurcation. This model thus provides a rare example of a system where a phase transition is caused by a non-local bifurcation, while both micro- and macro-level dynamics are accessible to mathematical analysis. The bifurcation points mark the onset of a behaviour that we call *network inoculation*. In the respective parameter region, exposure of the system to a pathogen will lead to an outbreak that collapses but leaves the network in a configuration where the disease cannot reinvade, despite every agent returning to the susceptible class. We argue that this behaviour and the associated phase transitions can be expected to occur in a wide class of models of sufficient complexity.

Throughout history, epidemic diseases have been a major cause of death in the human population. After a brief respite during the mid twentieth century, incidences of epidemics are now on the rise again, due to the emergence of new diseases such as Aids and Ebola, and the return of old killers, such as Tuberculosis and Influenza. Consequently, the study of epidemiology has received much recent attention from the mathematics and physics communities. In particular, network models provide a new theoretical tool by which the spreading of epidemic diseases can be understood and lessons for the real world can be learned. The present direction of this field is to push network models to greater realism by incorporating more and more aspects of real world epidemics, while maintaining mathematical and/or numerical tractability of the models. In this paper, we study the combined effect of two properties of real world contact networks across which real epidemics spread: adaptivity and heterogeneity. The network is adaptive in the sense that individuals in the network can respond to the presence of the disease, and it is heterogeneous in the sense that the individuals represented by network nodes have different properties, making them more or less susceptible to the disease. We show that combining these features leads to a phenomenon that we call *network inoculation*. Exposure of a given initial network to a pathogen can lead to an outbreak that collapses and leaves the network resistant to future outbreaks. This resistance is acquired solely through the rewiring of the network structure, without any nodes becoming physically immune to the disease. We use a variety of tools, including agent-based simulation, moment expansions, percolation methods, and numerical continuation, to reveal the heteroclinic mechanism that leads to this inoculation phenomenon.

## I. INTRODUCTION

A central goal in complex systems research is to understand how macroscopic transitions arise from the microscopic interactions within a system.^1^ In this context, an important role is played by coarse-grained models, describing the system in terms of a set of ordinary differential equations (ODEs).^2,3^ By capturing the dynamics of the system in terms of a suitable set of variables, it is sometimes possible to construct a faithful model of a given transition that is easy enough to be tractable by the tools of nonlinear dynamics. In the analysis, the transition then appears as a bifurcation, whose study reveals deep insights into the nature and behaviour of the underlying microscopic system.

A paradigmatic example is the epidemiological susceptible-infected-susceptible (SIS) model.^4^ In its simplest incarnation, this model describes the propagation of an infectious disease in a group of randomly interacting agents. Each agent is either infected with the disease (state I) or susceptible to the disease (state S). In time, the state of agents changes due to transmission of the disease and recovery of infected agents. The dynamics of this system can be understood by writing a single differential equation that captures the proportion of agents [*I*] that are infected. Depending on the details of interactions, the system either approaches a state where the disease is extinct or a state where it persists at a constant level. In the ODE-based model, the transition between the two qualitatively different types of behaviours occurs at a threshold parameter value that is a bifurcation point.

In the SIS model, and many other models besides, the important bifurcation is local, i.e., it is a bifurcation that can be characterised by changes in the phase portrait in the proximity of a single steady state or other invariant set.^5^ For instance, in the epidemic example, this bifurcation is a transcritical bifurcation in which a steady state with non-zero density of infected agents intersects the state where the disease is extinct, and the two exchange their stability. Thus, the relevant changes in the phase portrait occur in the vicinity of the extinct steady state.

The transcritical bifurcation and its close relatives, the fold and pitchfork bifurcations, have been linked to phase transitions in a wide variety of systems including epidemics,^4^ collective motion of animals,^6,7^ human opinion formation,^8,9^ neuronal dynamics,^10,11^ and others. In a smaller number of models, the underlying bifurcation is a Hopf bifurcation, which marks the onset of, at least transient, oscillations.^12–14^ However, even the Hopf bifurcation is a local bifurcation. By comparison, models in which a phase-transition corresponds to a non-local bifurcation in a macroscopic model are rare.

**FIG. 1.**
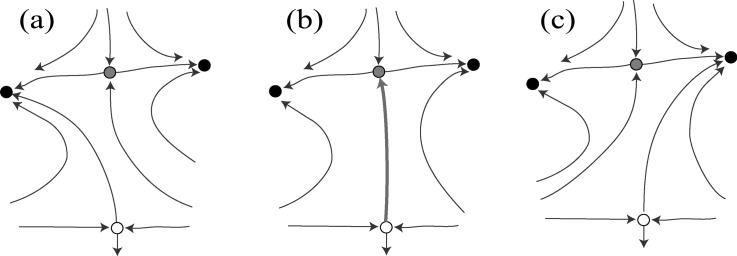
Sketch of the phase portrait before, during, and after a heteroclinic bifurcation. In the system, two attractors (black dots) coexist with two saddles (white and grey dots). The flow field is indicated by thin blue arrows. Before the bifurcation, a small perturbation launches the system on a trajectory leading to the left attractor (a). As parameters are changed, a heteroclinic connection between the saddles is formed, shown by the red strong arrow in (b). After the bifurcation, fluctuations on the white saddle can now lead to a final state at the right attractor (c), while the left attractor has become unreachable from the white saddle.

In nonlinear dynamics, several non-local bifurcations have been described. An example of particular interest for the present paper is the heteroclinic bifurcation.^15–17^ In this bifurcation, a transition in the macroscopic dynamics of a system occurs, due to the appearance of a trajectory connecting different invariant sets (see Fig. 1). Such bifurcations already occur robustly in relatively low-dimensional dynamical systems.^17^ The closely related homoclinic bifurcation often marks the point where a limit cycle is destroyed and thus causes a discontinuous phase transitions in many systems. One of these is the adaptive SIS model: an SIS system, where additionally the susceptible nodes try to avoid infection by rewiring their links away from infected nodes.^13^ In the adaptive SIS model, the importance of these homoclinic bifurcations is very minor but can play the role of an epidemic threshold in a small parameter space. Homoclinic bifurcations have also been observed in other network models, where information is exchanged globally between nodes, e.g., Ref. 18.

Homoclinics, heteroclinics, and other non-local bifurcations are also known to play a major role in fluid dynamics and climate system modelling.^19,20^ Perhaps, the best known example is the Lorenz model.^21^ Furthermore, heteroclinic orbits feature prominently in the analysis of switching in stochastic dynamics systems, where they describe optimal paths connecting regular saddles.^22,23^ However, these models are directly formulated on the macroscopic level, such that no direct connection to the phase transition in the underlying microscopic dynamics can be made. Evidence for such bifurcation was also seen in macro-level models of population dynamics.^24,25^ However, apart from a notable exception,^26^ models that resolve the detailed dynamics are often too complex to reveal a detailed picture of heteroclinics in the dynamics by use of bifurcation theory.

In a recent paper, we investigated the dynamics of a heterogeneous adaptive SIS model, which combined SIS dynamics and disease avoidance behaviour with heterogeneity in the susceptibility of the population. Both heterogeneity and adaptivity are known to impact the dynamics of diseases of humans^27^ and are therefore presently high on the agenda in network epidemiology. For instance, adaptivity was shown to significantly increase the epidemic threshold and lead to a first-order transition at the onset of the disease^28–32^ and can induce robust oscillations.^3^ Moreover, studies showed that adaptive disease avoidance behaviour can effectively enhance the impact of disease control efforts.^33–36^ The heterogeneity between individuals was shown to lower the epidemic threshold in some networks^37–39^ but can also reduce the size and risk of outbreaks.^39–44^

In Ref. 45, we found that a plausible disease avoidance mechanism can lead to states where the network has a heterogeneous topology but is more resilient to the invasion of diseases than it would be possible in less heterogeneous topologies. These findings are thus contrary to the intuition gained from landmark results for simpler models,^37,46^ which seem to suggest that heterogeneous topologies would always aid the transmission of the disease.

While our previous publication^45^ pointed to a mechanism that leads to the emergence of extraordinarily stable heterogeneous topologies, the actual transition at which this mechanisms sets in was too complicated to analyze within the scope of that paper. Here, we investigate this transition first in the previously proposed model and then in a highly stylized model that enables a deeper understanding of the phenomenon.

We find that the threshold for the onset of an endemic infection does not correspond to a loss of stability of the disease-free state. Instead, there is a large parameter range in which initial disease-free networks are unstable and thus permit disease invasion, but outbreaks do not lead to an endemic state but collapse back to another disease-free state, with different network topologies. The outbreak-and-collapse dynamics of the system in this region is thus reminiscent of an susceptible-infected-recovered (SIR) model. However, there is no recovered (R) agent state in the model that confers immunity. Instead, an initial outbreak leads to the formation of more resilient network topologies and thus “inoculates” the network against future disease invasion.

Network inoculation is characterized by the presence of heteroclinic orbits that connect different disease free states. Because of the basic physics of the system, the disease-free states form a manifold. As the infectivity of the disease is changed, the orbit starting from a given initial steady state connects to a (unique) saddle point. When this happens, a saddle-heteroclinic bifurcation occurs, which ends the inoculation-type dynamics from the respective initial network. For all higher values of infectivity, the heteroclinic trajectory from that initial state leads to an endemic state where the disease can persist in the system indefinitely. Thus, the onset of endemic disease dynamics is marked by a phase transition caused by a heteroclinic bifurcation in the underlying dynamics.

This paper is organized as follows: We start by reviewing the previously proposed model (Sec. II). In agent-based simulations, we observe that the outcomes of simulation runs can be classified into 3 different types (Sec. III). We then explore the phase boundaries between the three different types of outcomes. Using percolation theory, we analytically compute the threshold where outbreaks start to occur (Sec. IV). Thereafter, using moment expansions, we formulate a macroscopic model of the dynamics in terms of ordinary differential equations (Sec. V), and this model allows us to study the dynamics by tools of dynamical systems theory. Combining results from all of the tools established up to this point, we show that the transition from outbreaks to endemic behavior occurs due to a heteroclinic bifurcation (Sec. VI). To understand this transition in greater detail, we finish by formulating and analyzing a simpler solvable model for the network inoculation phenomenon (Sec. VII).

## II. HETEROGENEOUS ADAPTIVE SIS MODEL

We consider a population of *N* agents, which can be either infected (state I) or susceptible to the disease (state S). The agents are connected by a total of *K* bidirectional social contacts. Thus, the system can be described as a network in which the agents are the network nodes and the social contacts are the links. In time, the system evolves (a) because of the epidemic dynamics and (b) due to a behavioural response of the agents to the disease, which leads to the rewiring of links.

In the epidemic dynamics (a) for every link connecting a susceptible and an infected agent, there is a chance that the susceptible agent becomes infected, amounting to an infection rate of βψ (per link), where *β* is a parameter that controls the overall infectivity of the disease and *ψ* is a parameter that describes the susceptibility of the susceptible agent. In particular, we consider the case where two types of agents exist: highly susceptible agents (type A) and less susceptible agents (type B). These types are intrinsic properties of the agents, i.e., unlike the epidemic states the type of an agent never changes. Furthermore, all infected agents recover at a fixed rate *μ*, which is identical for all agents. Upon recovery, agents immediately become susceptible again.

We denote the proportion of agents of type A in the population by $p_{a}$ and their susceptibility by $\psi_{a}$. The remaining portion of agents $p_{b}=1-p_{a}$ is of type B and has susceptibility $\psi_{b}<\psi_{a}$. In the following, we chose these parameters such that $p_{a}\psi_{a}+p_{b}\psi_{b}=\langle \psi \rangle =0.5$. We thus control the heterogeneity of susceptibility in the population by changing $\psi_{a}$ and $\psi_{b}$ simultaneously such that the mean susceptibility ⟨ψ⟩ remains fixed. Hence, the intra-individual heterogeneity is indicated by one of the parameters, say, $\psi_{a}$, whereas the overall spreading rate is controlled by the epidemic parameter *β*.

In the social dynamics (b), the agents react to the presence of the disease by rewiring their social connections. In each small time interval of length *dt*, a susceptible agent who is linked to an infected agent breaks that link with probability *ωdt*. For every link a susceptible agent breaks, the agent establishes a new link to a randomly chosen susceptible agent, such that the total number of links is conserved.

In the following, we use the parameters $N=10^{5},K=10^{6},\;\omega =0.2,\;\mu =0.002$, and ⟨ψ⟩=0.5 unless noted otherwise. Hence, the mean degree of the network is ⟨k⟩=2K/N=20. This is a typical parameter set where the model shows its generic behaviour. In our experience, the results obtained are very similar for other parameter sets unless extreme values are chosen.

## III. CLASSIFICATION OF OUTCOMES

We start the analysis by numerically exploring the possible outcomes in agent-based simulations. We initialize the system as an Erdős-Rényi random graph, where the number of agents *N* and links *K* is fixed and mean degree is then given by ⟨k⟩=2K/N. Each agent (regardless of type) is initially infected with probability $i_{0}=0.0002$ and susceptible otherwise. We then simulate the time evolution of the system of agents using a Gillespie algorithm.^47,48^

**FIG. 2.**
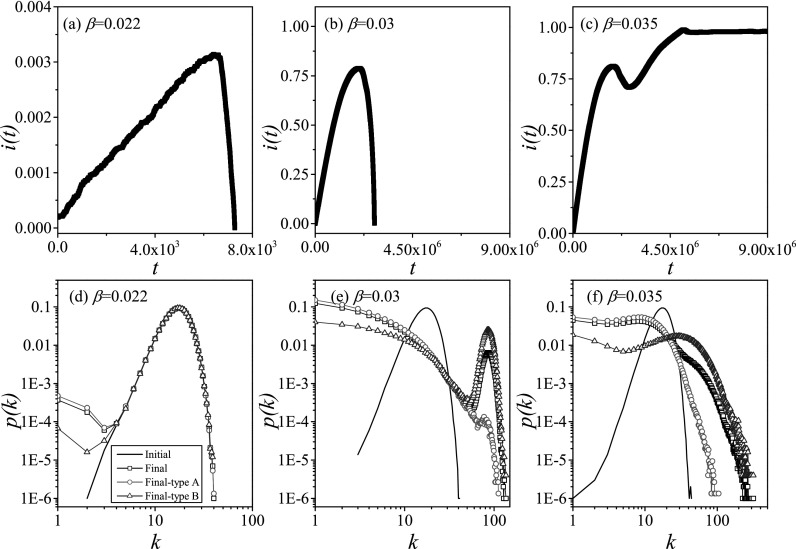
Three simulation results from agent-based simulations. Top row: If the infectivity is low (left, β=0.022), then the epidemic dies out quickly and the system freezes in the disease-free state (note the different axis scalings on this plot). For intermediate infectivity (center, β=0.03), there is an initial outbreak, which infects a large proportion of the agents. However, subsequently this outbreak collapses and the system once again approaches the disease free state. If infectivity is high (right, β=0.035), then the system approaches an endemic state where the diseases remain in the system in the long term. Bottom row: Degree distributions in the final state (symbols) in comparison to the initial degree distribution (line). If the infectivity is low (left, β=0.022), the disease has only a very minor impact on the degree distribution due to its quick collapse. For intermediate infectivity (center, β=0.03), the rewiring leads to a bimodal distribution where many links are concentrated on the nodes of the less susceptible type, which prevents further outbreaks. If infectivity is high (right, β=0.035), ongoing rewiring leads to a broad continuous distribution, but this is no longer sufficient to lead to a collapse of the epidemic. Parameters: $\psi_{a}=0.65,\;\psi_{b}=0.05,\;\omega =0.2,\;\mu =0.002,\;i_{0}=0.0002,\;N=10^{5},\;K=10^{6}$.

Three typical outcomes are shown in Fig. 2. Depending on the parameter values, we observe either a rapid collapse to a disease-free state, before a significant proportion of the agents have been infected (type I), an initial epidemic outbreak, in which a large proportion of agents are infected (type II), or an outbreak leading to an endemic state where the disease persists indefinitely (type III).

Let us try to extrapolate from the finite-size simulation to arbitrarily large systems. The results of this analysis should hold in large finite systems encountered in the real world or studied in large agent-based simulations, where finite size effects are mostly irrelevant, due to the size of the system considered. Moreover, referring to an infinitely large system is attractive because it allows us to avoid problems in the classification of behaviours that exist in the finite system. Consider that in the finite case the difference between type-I (recovery to the disease-free state) behaviour and type-II (outbreak, collapse) behaviour is not rigorously defined, i.e., the transition is gradual as the number of infected at maximum increases. Furthermore, even the difference between type-II and type-III (persistent) behaviour becomes fuzzy: The finite size agent-based simulation has a finite probability to spontaneously collapse to the absorbing disease-free state. Thus, persistent dynamics cannot be a true long-term behaviour, although we never observed such a collapse of apparently persistent epidemics in all but the smallest simulation runs (e.g., *N* < 100) or when the system is just at the epidemic threshold.

By contrast, the different types of behaviours can be cleanly defined in the infinite system. We say, that the behavior of the system is of type I, if the epidemic never grows to a point where a finite proportion of the agents is infected. This makes type-I behavior qualitatively different from type II and type III, where at some point a finite proportion of the agents is infected. We further distinguish type-II and type-III behaviours by their long-term behavior: We can say, that a system shows type-III behavior if in the infinite size limit, a finite proportion of the agents are infected after arbitrarily long time.

**FIG. 3.**
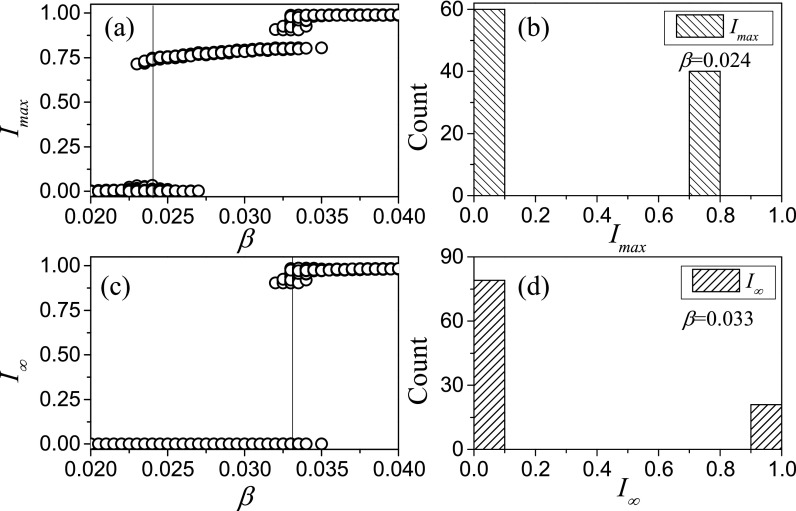
Classification of outcomes from agent-based simulations. Shown are the maximal proportion of infected agents encountered in a simulation run, $I_{\text{max}}$ (top left) and the proportion of infected after long time *t*, $I_{\infty }$ ($t=10^{7}$, bottom left). The symbols represent observed outcomes for each of 100 simulation runs for each value of infectivity *β*, many of which are so similar that they are indistinguishable. It is apparent that three qualitatively different outcomes are observed: $I_{\infty }\approx 0,I_{\text{max}}\approx 0$ (type I), $I_{\infty }\approx 0,\;I_{\text{max}}>0$ (type II), and $I_{\infty }>0,\;I_{\text{max}}>0$ (type III). While two different outcomes are possible for some values of *β*, they can be clearly distinguished in this case, see Histograms in the panels on the right, with values of *β* corresponding to the thin lines shown in the left plots. Parameters: $\psi_{a}=0.65,\;\psi_{b}=0.05,\;\omega =0.2,\;\mu =0.002$, $i_{0}=0.0002,\;N=10^{5},\;K=10^{6}$.

Now returning to finite systems, the considerations above enable us to classify the dynamics using scaling relationships. However, in practice, this is not necessary as the differences in sufficiently large simulations are clear cut. Results from simulations with $N=10^{5}$ nodes in Fig. 3 show that the three types of outcomes can be clearly distinguished. We note that none of the simulation runs has $I_{\text{max}}\in (0.1,0.6)$ or $I_{\infty }\in (0.1,0.75)$. Thus, choosing thresholds anywhere in these ranges will lead to the same classification of outcomes.

**FIG. 4.**
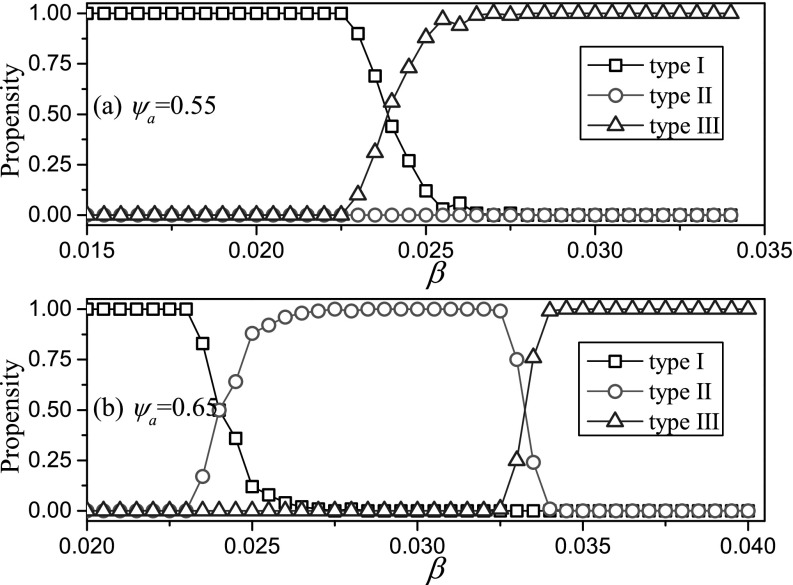
Propensity of outcomes depending on infectivity (*β*) and heterogeneity ($\psi_{a}$). Shown is the probability that a given type of behavior is observed when simulating a random initial network with the respective parameter values (see text). These probabilities were estimated by classifying the outcomes of 100 simulation runs for each parameter combination. For low values of heterogeneity (top, $\psi_{a}=0.55$), we observe type-I (recovery) behavior if infectivity is low and type-III (endemic) behavior if infectivity is high. At intermediate values, there is a transition region where both outcomes are possible. For systems with strong heterogeneity (bottom, $\psi_{a}=0.65$), additionally type-II (outbreak, collapse) behavior is observed at intermediate values of infectivity, which is separated from type-I and type-III behavior by two transition regions. Parameters: $\psi_{b}=0.05,\;\omega =0.2,\;\mu =0.002,\;i_{0}=0.0002,\;N=10^{5},\;K=10^{6}$.

We observe that in some ranges of infectivity, different types of outcomes are possible. The regions in which different outcomes are possible seem to overlap, and around β=0.33 there is heterogeneity in the prevalence of the endemic state. The latter is a numerical effect which appears due to intrinsic stochasticity of the agent based simulation, which leads to different amounts of rewiring during the approach to the endemic state. The mechanism that causes these differences between trajectories will become apparent in Sec. VI. To explore the former effect in more detail, we use the proposed classification to plot the propensity of outcomes in Fig. 4. For low values of heterogeneity between nodes $\psi_{a}=0.55$, we find that for systems there are only two possible outcomes, namely, type-I (recovery) and type-III (endemic) behavior. However, if the susceptibility of agents is very heterogeneous, then also type-II (outbreak, collapse) behavior is observed.

**FIG. 5.**
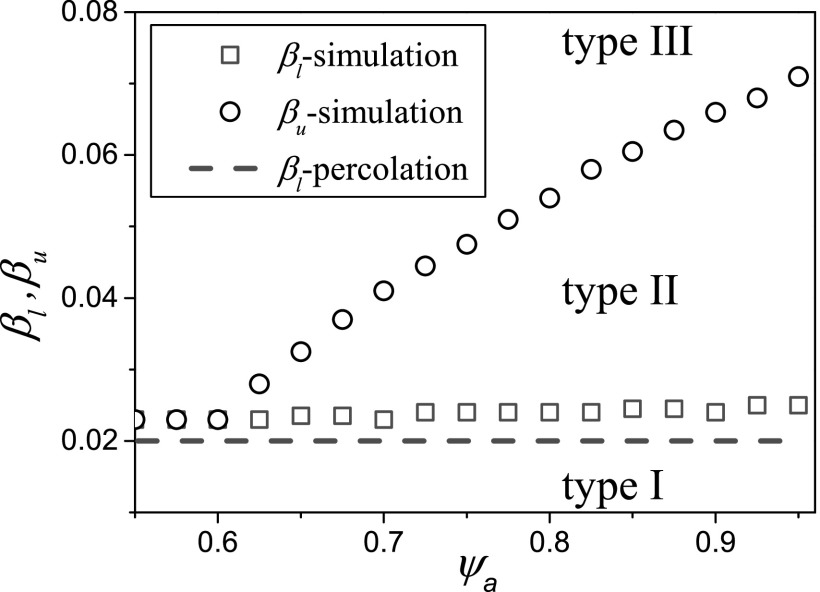
Phase boundaries between different types of outcomes: *β_l_* refers to the I/II boundary and *β_u_* to the II/III boundary. Shown are results from the classification of simulation runs (symbols) and an estimate using percolation theory from Eq. (5) (dashed line). Parameters: $\psi_{b}=0.05,\;\omega =0.2,\;\mu =0.002,i_{0}=0.0002,\;N=10^{5},\;K=10^{6}$.

In Fig. 4, we see that regions of different types of outcomes are separated by transition regions where 2 outcomes are possible. To prepare for the more detailed exploration below, let us now construct a 2-parameter phase diagram of the system (Fig. 5). In this diagram, we draw the phase boundaries at the points where different types of outcomes occur in simulation, e.g., the phase boundary between outcomes of type I and type II *β_l_* is set of points where the type-II outcome starts to show up and the same to the phase boundary between type II and type III *β_u_*.

## IV. ONSET OF OUTBREAKS

Let us now try to understand the phase diagram analytically. We start by considering the onset of outbreaks, i.e., the boundary of type-I behaviour. The ability of a disease to spread in a population can be quantified in terms of the basic reproductive number *R*_0_, which denotes the number of secondary infections, caused by one infected, in the limit of low disease prevalence. If $R_{0}>1$, the disease can percolate through the network and thus outbreaks become possible.

We can compute *R*_0_ by considering a typical newly infected agent and computing the number of neighbours this agent will infect before recovering. Following Ref. 13, we take into account that the number of links of the focal agent decreases in time as neighbouring agents rewire away. The loss rate of links is equal to the rewiring rate *ω*. Thus, the remaining degree after time *t* is

$$k(t)=k_{0}e^{-\omega t},$$ (1)

where *k*_0_ is the initial number of neighbours. Since we are interested in the limit of low prevalence, all neighbours can be assumed to be susceptible and we can find the number of secondary infections by multiplying the probability of transmission, which we call *p* for the moment, and then integrating over the typical time to recovery 1/μ. This yields

$$R_{0}=p\int_{0}^{1/\mu }k_{0}e^{-\omega t}\text{dt}=\frac{pk_{0}}{\omega }\left(1-e^{-\frac{\omega }{\mu }}\right).$$ (2)

For the heterogeneous network, we can express the probability of transmission *p* as

$$p=\beta (x_{a}\psi_{a}+x_{b}\psi_{b}),$$ (3)

where *x_a_* is the probability that a randomly chosen neighbour is of type A and *x_b_* is the probability that a randomly chosen neighbour is of type B. As the initial network is an Erdős-Rényi random graph, *x_a_* = *p_a_*, *x_b_* = *p_b_*, and $k_{0}=\langle k\rangle$. Substituting into Eq. (2) and setting $R_{0}=1$ yields

$$1=\frac{\beta (p_{a}\psi_{a}+p_{b}\psi_{b})\langle k\rangle }{\omega }\left(1-e^{-\frac{\omega }{\mu }}\right),$$ (4)

and hence the threshold

$$\beta_{l}=\frac{\omega }{\langle k\rangle \langle \psi \rangle \left(1-e^{-\omega /\mu }\right)},$$ (5)

with $\langle \psi \rangle =p_{a}\psi_{a}+p_{b}\psi_{b}$. Expectedly, this equation is very closely related to the epidemic threshold in the homogeneous system. The two values of *ψ* are effectively averaged and only the numerical mean appears.

A comparison of the outbreak threshold identified based on percolation arguments and the numerical results show good qualitative agreement (Fig. 5). In the simulations, we observe the outbreak only at slightly higher levels of infectivity, which is most likely a finite size effect. Closely above to the theoretical threshold for the infinite size system, the finite size simulation can still collapse to the absorbing disease free state due to stochastic extinction.

The results obtained above were based on the assumption that agents of type A and type B are well mixed. While this assumption is true in the initial state, very different outbreak thresholds can be found if the assumption is violated, for instance, if rewiring in response to an earlier outbreak led to a non-random mixing in the population. We explore this particular scenario in detail in Section V.

To gain a general understanding of the effects of assortativity in the disease free state, let us now consider a disease free state with given number of a–a and b–b links. We denote the density of these links in the population by [aa] and [bb], respectively. The numerical values of both of these quantities are understood to be normalized with respect to the total number of nodes *N*. In this notation, the density of a–b links [ab] can then be computed from the conservation law

⟨k⟩=2([aa]+[ab]+[bb]). (6)

Given [aa] and [bb], we can therefore write the number of nodes of types *i* that are infected by a given node of type *j* as

$${R\prime }_{i,j}=\frac{\beta \psi_{i}[ij](1+\delta_{i,j})}{\omega p_{j}}\left(1-e^{-\frac{\omega }{\mu }}\right).$$ (7)

These values form the entries in a 2 × 2 next-generation matrix. The disease can spread if the leading eigenvalue of this matrix is larger than one. By pulling the repeated factor out of the matrix, we get the condition

$$\lambda >\frac{\omega }{\beta \left(1-e^{-\frac{\omega }{\mu }}\right)},$$ (8)

where *λ* is the leading eigenvalue of

$$R\prime =\left(\begin{array}{cc} \frac{2\psi_{a}[aa]}{p_{a}} & \frac{\psi_{a}[ab]}{p_{b}}\\ \frac{\psi_{b}[ab]}{p_{a}} & \frac{2\psi_{b}[bb]}{p_{b}} \end{array}\right).$$ (9)

**FIG. 6.**
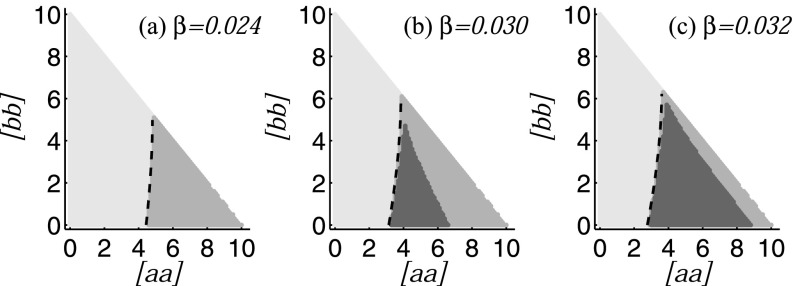
Impact of the network structure in the initial state. Shown is the stability threshold found by percolation methods, Eq. (8) (dashed line), in comparison to local asymptotic stability of the disease-free state computed based on the eigenvalues of the Jacobian matrix of the moment equations (Sec. V). The figure shows the regions of stable disease free (type I, light grey), outbreak and collapse (type II, medium grey), and endemic (type III, dark grey) behavior, where we used numerical integration of the moment equations to distinguish between types II and III. In the remainder of the figure (white), no networks exist as the sum of a–a links and b–b links would be greater than the total number of links in the system. The figure shows that the agreement between the threshold for the onset of outbreaks computed by the two different approximations is almost perfect. Parameters: $\psi_{b}=0.05,\;\psi_{a}=0.65,\;\omega =0.2,\;\mu =0.002$, $i_{0}=0.0002,\;N=10^{5},\;K=10^{6}$, and [aa]+[ab]+[bb]=⟨k⟩/2.

This provides a condition that can be solved for, say, the critical number of a–a links [aa] at which outbreaks start. While easy to compute, this condition is quite lengthy and is hence omitted here. The result is shown in Fig. 6.

The computation shows that for a given value of infectivity, an outbreak can occur if the density of a–a links is sufficiently high. This is intuitively reasonable as a disease close to the threshold will mainly spread in the highly susceptible (type A) population.

## V. MOMENT EXPANSIONS

Percolation approaches, such as the ones above, are powerful tools for exploring the onset of the epidemic. However, these approaches cannot reveal insights into the dynamics that occur after the onset. We therefore have to switch to a different modelling approach. Here, we use network moment expansions. Following the procedure in Refs. 13 and 45, we write a system of differential equations that capture the dynamics of the abundances of different types of links and node states. We use symbols of the form $\left[X_{u}\right]$ and $\left[X_{u}Y_{v}\right]$ with X,Y∈{I,S} and u,v∈{a,b} to, respectively, denote the proportion of agents and per capita density of links between agents of a given type. For instance, $\left[I_{a}\right]$ is the proportion of agents that are infected and of type A, and $\left[S_{a}I_{b}\right]$ is density of links between susceptible agents of type A and infected agents of type B. All of these variables are normalized with respect to the total number of nodes *N*. Given the number of infected nodes of a given type, we can thus find the number of susceptible nodes by using the conservation law $[I_{u}]+[S_{u}]=p_{u}$.

The time evolution of the proportion of nodes that are infected and of types A and B can be, respectively, written as

$$\frac{d}{\text{dt}}\left[I_{a}\right]=-\mu [I_{a}]+\beta \psi_{a}\sum_{v}[S_{a}I_{v}],$$ (10)

$$\frac{d}{\text{dt}}[I_{b}]=-\mu [I_{b}]+\beta \psi_{b}\sum_{v}[S_{b}I_{v}].$$ (11)

For the link densities, using a pair-approximation leads to equations of the form

$$\begin{array}{c} \frac{d[S_{a}S_{a}]}{dt}=\mu [S_{a}I_{a}]-2\beta \psi_{a}(\frac{\left[S_{a}S_{a}][S_{a}I_{a}\right]}{\left[S_{a}\right]}+\frac{\left[S_{a}S_{a}][S_{a}I_{b}\right]}{\left[S_{a}\right]})+\frac{\omega [S_{a}]}{\left[S_{a}]+[S_{b}\right]}([S_{a}I_{a}]+[S_{a}I_{b}]), \end{array}$$ (12)

where the terms on the right-hand-side describe the impact of the different processes on the motif considered, $\left[S_{a}S_{a}\right]$ in this example. For instance, the first term corresponds to the creation of $S_{a}$–$S_{a}$ links due to recovery of the infected node in $S_{a}$–$I_{a}$ links. In total, the $I_{a}$ nodes recover at the rate $\mu [I_{a}]$. Every such recovery event creates an expected number of $S_{a}$–$S_{a}$ links that is identical to the average number of $I_{a}$–$S_{a}$ links anchored on an $I_{a}$ node, which is $\left[I_{a}S_{a}]/[I_{a}\right]$. In summary, the change in the density of $S_{a}$–$S_{a}$ links due to recovery of $I_{a}$ nodes is $\mu [I_{a}][I_{a}S_{a}]/[I_{a}]=\mu [I_{a}S_{a}]$, which explains the first term in Eq. (12). In addition to the equation shown above, there are 8 other differential equations capturing the density of other types of links. For conciseness, these equations are shown in the Appendix.

**FIG. 7.**
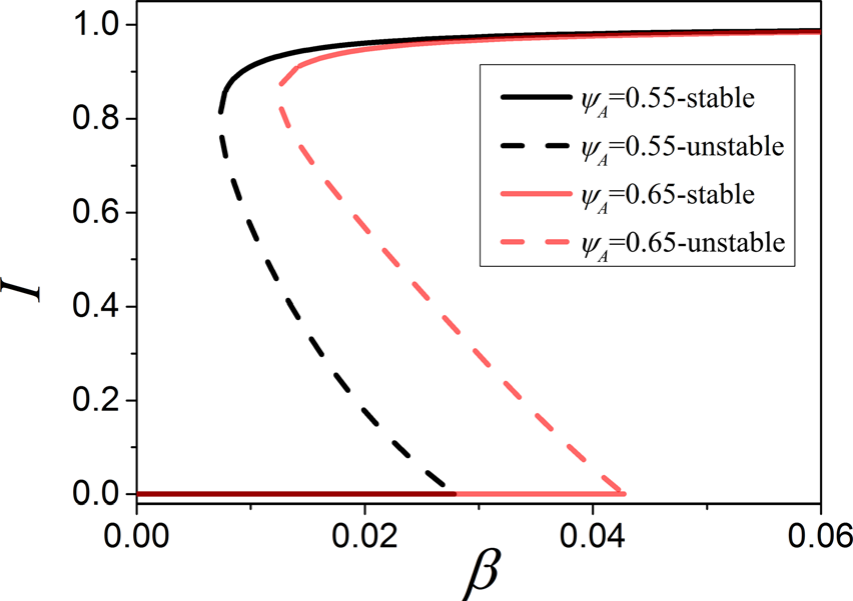
Bifurcation diagram of the moment equations. Shown is the stationary disease prevalence *I* as a function of infectivity *β* for two values of heterogeneity, $\psi_{a}=0.55$ (black) and $\psi_{a}=0.65$ (red/gray). Numerical continuation reveals both stable (solid) and unstable (dashed branches). Stability changes due to a transcritical (TC) and saddle-node bifurcations (SN). Between these two bifurcations, a hysteresis loop is formed that is typical for adaptive SIS models. Parameters: $\psi_{b}=0.05,\;\omega =0.2,\;\mu =0.002,N=10^{5},\;K=10^{6}$.

In contrast to the percolation approach and agent-based simulations, the moment expansion allows us to investigate the dynamics directly on an emergent level. In the context of the moment equations, the different types of long-term behaviours now appear as attractors of a dynamical system. We analyze this system by numerical continuation of solution branches in AUTO.^49^ Results from this analysis are shown in Fig. 7.

At sufficiently high infection rate, there is a stable steady state where the disease persists with high prevalence. When we gradually lower the infection rate, this steady state becomes unstable due to a saddle-node bifurcation, or by undergoing a Hopf bifurcation quickly followed by saddle-node bifurcation, depending on parameters. The limit cycle formed in the Hopf bifurcation only exists in a very small parameter range before it is destroyed in further bifurcations.

The situation is more complex for the disease free states. While the branches of steady states where the disease is present have well-defined values in all of the dynamical variables, the disease free states form a manifold. All states in which the density of infected nodes is zero are necessarily stationary. However, this still permits networks with different values of the variables [aa] and [bb].

Above we already explored the stability of the manifold of disease-free steady states using the microscopic branching process approach. We can now replicate these results using the macroscopic moment expansion approach. For this purpose, we compute the Jacobian matrix of the moment equations on the manifold of the disease-free steady states. These states are then stable if the leading eigenvalue of the Jacobian has a negative real part. A comparison of the threshold that is thus obtained with the previous results (see Fig. 6) shows that the two approaches are in almost perfect agreement. We have furthermore verified by direct simulation of the agent-based model that introduction of the disease does not cause outbreaks in networks where the disease-free state is predicted to be stable.

## VI. TRANSITION TO THE ENDEMIC STATE

Let us now turn our attention to the transition between type-II and type-III behavior. In Secs. IV and V, we have investigated the local bifurcations of the initial state and endemic states. The results show that in sufficiently heterogeneous systems, the local bifurcations of these states do *not* coincide with the phase transition to endemic behavior: This initial state is unstable and the endemic state is stable before the onset of type-III behavior. This is possible because the initial state is not in the basin of attraction of the endemic state. Starting from the initial state, the system undergoes a single outbreak before it falls back to a different disease-free state (with a different distribution of links between node types) which is then stable against further outbreaks.

The transition between type-II (single outbreak) and type-III (endemic state) behavior is represented by a transition of the initial disease-free saddle from one basin of attraction to another. For a given parameter set, we can visualize three different basins of attraction (Fig. 6). We note that type-II (outbreak-collapse) behavior occurs when the density of a–a links is high, whereas endemic behavior is observed for intermediate density of a–a links. While the a–a link density has to exceed a threshold value to allow outbreaks, the outbreak eventually collapses if a second threshold is exceeded.

We note that outbreak (type II) dynamics always land the network in a final state that is characterized by a lower connectivity of the highly susceptible type A nodes, in which disease propagation is suppressed. We verified this observation both in the moment equations and by agent-based simulation.^45^ Hence, one can say, that the outbreak inoculates the network against subsequent outbreaks of the same disease.

**FIG. 8.**
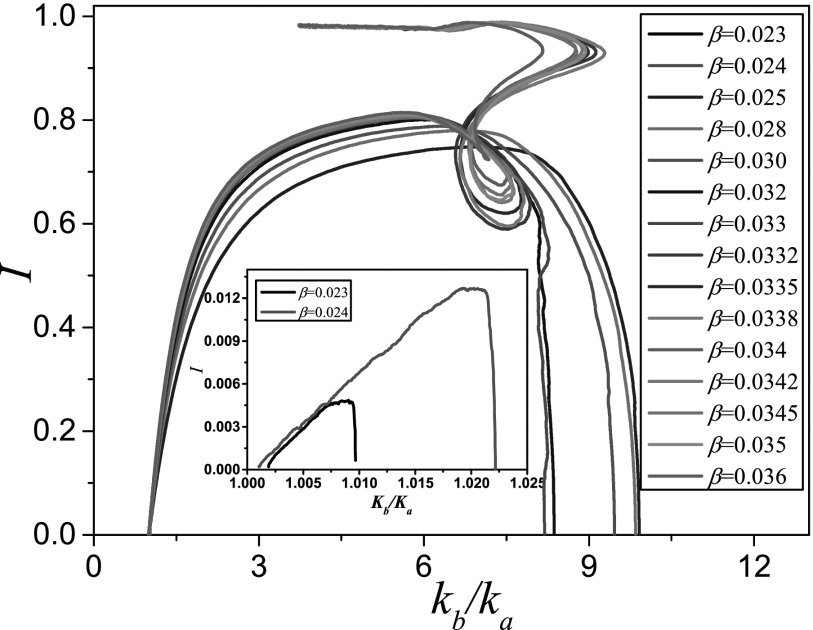
Trajectories from agent-based simulation. Shown are 15 trajectories starting from the same initial state at different values of infectivity *β*. At low infectivity, the trajectories remain in the vicinity of the initial state (inset). At higher infectivity, there is an initial outbreak leading to high values of prevalence *I* before collapsing back to a disease free state, where the ratio between the degree of type B and type A nodes is now much higher than in the initial network. At even higher values of infectivity, endemic behavior is observed as the system approaches a stable state with high prevalence. The transition to endemic behaviour occurs when trajectories encounter a point where the dynamics is almost stationary, which points to a heteroclinic bifurcation. Parameters: $\psi_{a}=0.65,\;\psi_{b}=0.05,\;\omega =0.2$, $\mu =0.002,i_{0}=0.0002,N=10^{5},\;K=10^{6}$.

In the transition from type-II to type-III behaviour, the saddle point that is the initial state leaves the basin of attraction of the stable disease-free state and enters the basin of attraction of the endemic state (Fig. 8). For such a transition of a saddle from one basin to another, a heteroclinic bifurcation is a likely candidate. Moreover, as the parameter is tuned closer to the transition point, the trajectories start to approach the saddle point that is formed in the fold bifurcation of the endemic state (see Fig. 7). In Fig. 8, one can see one of the trajectories turning sharply in as it passes close to the saddle. This shows the transition between type-II and type-III behaviour, which provides further evidence that the transition is caused by a saddle-heteroclinic bifurcation. In this bifurcation, the stable manifold from the saddle hits the initial state, such that a heteroclinic connection between saddles is formed. This connection also marks a basin boundary, such that in the bifurcation the initial state passes from one basin of attraction to the other.

**FIG. 9.**
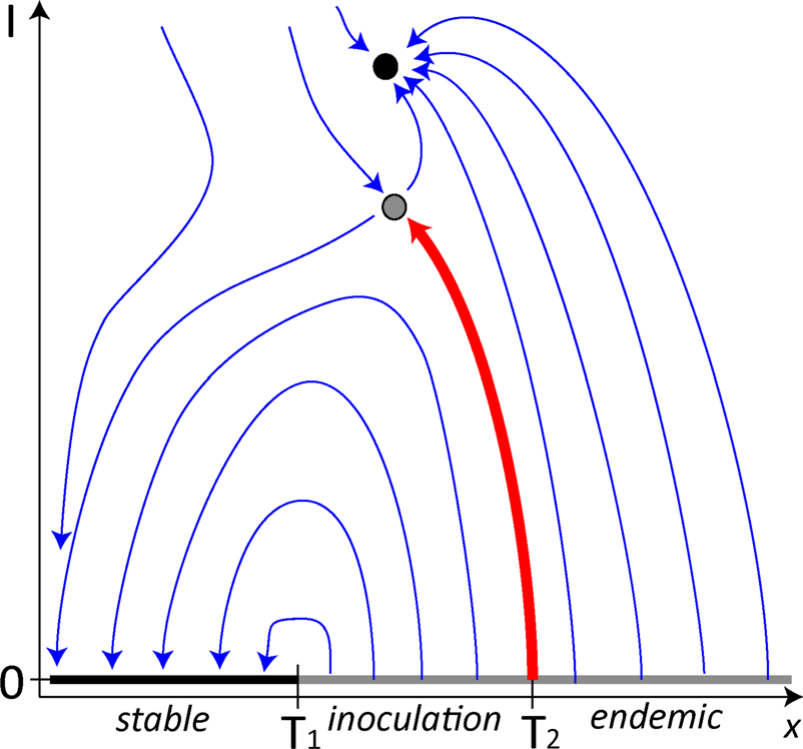
Simplified sketch of the phase portrait in the epidemic model. Shown is a flow field (thin blue arrows), the attracting endemic state (black circle), a saddle point (grey circle) and a manifold of disease-free steady states (strong grey/black line), which can be stable (black) or unstable (grey). Depending on the initial value of the x-axis, we can distinguish between stable disease-free (type-I), outbreak and collapse (type-II), and endemic (type-III) behavior, indicated by labels on the axis. The behaviour changes at two threshold values (*T*_1_, *T*_2_) which are marked by a local change in the stability of the manifold and the heteroclinic connection. We note that this sketch has been simplified from the situation in the epidemic model. If the x-axis was the a–a link density [aa], the type-II behavior would occur for intermediate values, whereas the type-III behavior would occur at high values, which is harder to visualize in a 2d-plot, but qualitatively similar.

We can illustrate the situation with a simplified sketch of the phase portrait (Fig. 9). The figure shows how two thresholds divide the manifold of disease-free steady states into different sections in which perturbations lead to three different types of outcomes observed. If other parameters of the system change, then these two thresholds move such that for a given initial condition, the transitions appear as transcritical and heteroclinic bifurcations, respectively.

Let us emphasize that the x-axis in Fig. 9 cannot be the variable [aa] as the different types of behaviour would occur in a different order (cf. Fig. 6). The different order of sections when plotted over [aa] does not imply qualitatively different dynamics but is more difficult to visualize in a two-dimensional sketch.

## VII. SOLVABLE STYLIZED MODEL

Even the simplified ODE system discussed above has eleven degrees of freedom, and as such it is difficult to analyse in detail. However, the basic phenomenon of inoculation via a heteroclinic bifurcation can be captured in a solvable two-dimensional stylized model as we now describe. We consider a well-mixed population with two susceptible types (denoted *S_a_* and *S_b_* as previously) and a single infective type *I*. To see inoculation without resolving the network structure, it is necessary to introduce a new non-linear term to induce bistability. We keep the same infection as above but make a modification to recovery: instead of spontaneous recovery, infectious individuals may be coopted back to a susceptible state by interaction with a pair of susceptible individuals of the same type.

While the cooption to the susceptible type may seem strange at first glance, very similar mechanisms are typically considered in threshold models of opinion formation, including, for instance, an adaptive network model for opinion formation among locusts.^7^ While we intend the proposed model mainly as an abstract illustration, one can imagine that very similar models can be relevant in situations where both opinion formation and epidemic processes occur. This is the case, for instance, when choices can be made that prevent infection (e.g., vaccination) or transmission (e.g., hygiene and safer sex).

The dynamics of the simplified model are captured by the rate equations

$$\begin{array}{c} \frac{d[S_{a}]}{dt}=-\beta \psi_{a}[I][S_{a}]+\mu [I]{\left[S_{a}\right]}^{2},\\ \frac{d[S_{b}]}{dt}=-\beta \psi_{b}[I][S_{b}]+\mu [I]{\left[S_{b}\right]}^{2},\\ \frac{d[I]}{dt}=\beta [I](\psi_{a}[S_{a}]+\beta \psi_{b}[S_{b}])-\mu [I]({\left[S_{a}\right]}^{2}+{\left[S_{b}\right]}^{2}). \end{array}$$ (13)

Note that the system is two-dimensional since $[S_{a}]+[S_{b}]+[I]=1$ is a conserved quantity. The line [I]=0 is a manifold of fixed points. Along the absorbing lines $[S_{a}]=0$ or $[S_{b}]=0$, the system is reduced to the one-dimensional ODE

$$\frac{d[I]}{dt}=\beta \psi_{*}[I](1-[I])-\mu [I]{\left(1-[I]\right)}^{2}\;,$$ (14)

where ∗∈{a,b}. The form of this equation already suggests the existence of a heteroclinic bifurcation. There are always steady states at [I]=0 (extinction) and [I]=1 (endemic infection), with the possibility of a third at $[I]=1-\beta \psi_{*}/\mu$. If this third steady state lies in (0, 1) then it is a saddle, and the extinct and endemic states are stable. If it lies outside the physically relevant region, then the extinct state is unstable.

By choosing $\psi_{b}<\psi_{a}$ appropriately, we are able to realise a situation in which there is a saddle on the $[S_{a}]=0$ line but not on $[S_{b}]=0$. This structure motivates the unusual non-linear choice made for recovery.

**FIG. 10.**
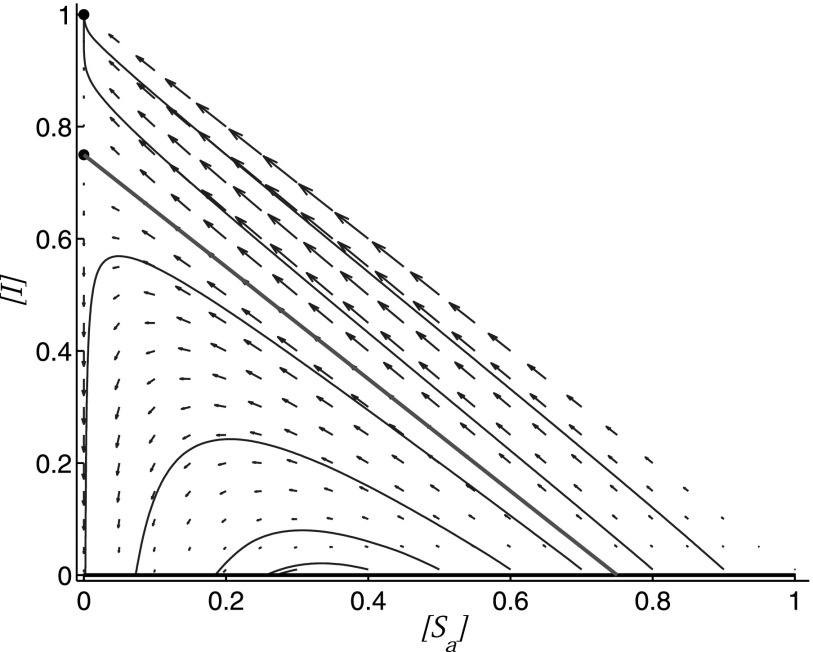
Phase portrait for the simplified model. The phase portrait contains a manifold of steady states (strong black line) at zero prevalence. In addition, there are two steady states at non-zero prevalence (black dots). The lower of these two states is a saddle whose stable manifold (red line) forms the separatrix between outbreak and endemic behavior. This is illustrated by the flow field (blue arrows) and example trajectories (thin blue lines). Parameters: $\beta =0.5,\;\mu =0.5,\;\psi_{a}=1,\;\psi_{b}=0.25$.

The phase portrait of the system is shown in Fig. 10. From the figure, perturbation around a state with [I]=0 has three possible outcomes. For small $\left[S_{a}\right]$, we have a type-I region, where no outbreaks can occur. For large $\left[S_{a}\right]$, the trajectory is carried all the way to the stable endemic equilibrium at [I]=1 in a type-III scenario. In between, there is a range of values for $\left[S_{a}\right]$ with type-II trajectories that initially depart but then return to the [I]=0 line. This region is bounded on the left by the point where the non-zero eigenvalue of the Jacobian matrix changes sign, which we compute to be the point where $\left[S_{a}\right]$ solves

$$0=\beta \psi_{a}[S_{a}]+\beta \psi_{b}(1-[S_{a}])-\mu {\left[S_{a}\right]}^{2}-\mu {\left(1-[S_{a}]\right)}^{2}\;.$$ (15)

On the right, the type-II region is bounded by the separatrix of the endemic and extinct states, which can be found by examining

$$\frac{d[I]}{d[S_{a}]}=-1+\frac{1-[I]-[S_{a}]}{\left(-\beta \psi_{a}+\mu [S_{a}])[S_{a}\right]}(-\mu (1-[I]-[S_{a}])+\beta \psi_{b})\;,$$ (16)

implying the separatrix $[I]=1-[S_{a}]-\beta \psi_{b}/\mu$.

**FIG. 11.**
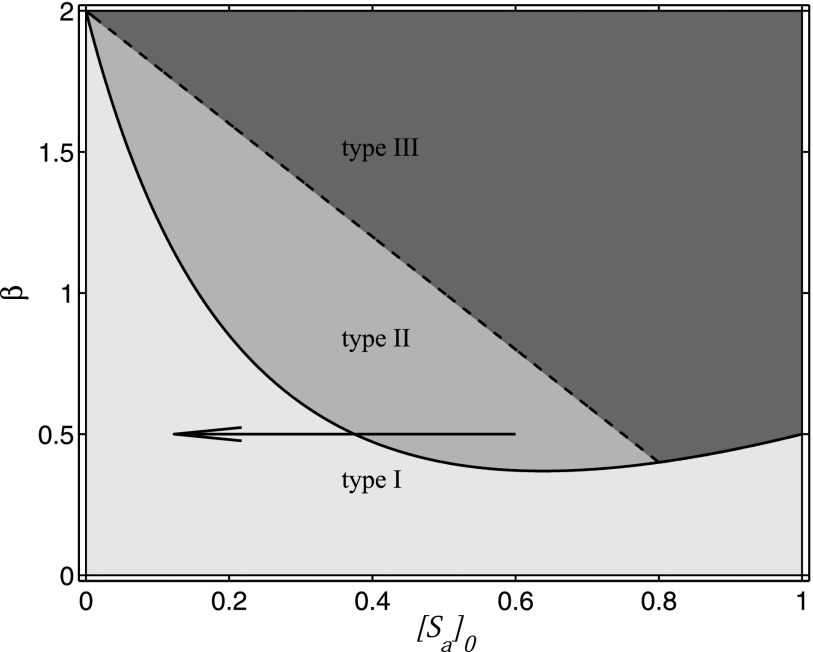
Phase diagram of the simplified model. Transcritical (solid line) and heteroclinic (dashed line) bifurcation separated phases of qualitatively different behaviors: type I (disease free, light grey), type II (outbreak and collapse, medium grey), and type III (endemic, dark grey). Outbreaks take the system from the type-II region into the type-I region (black arrow) and thus inoculate it against further outbreaks. Parameters: $\mu =0.5,\;\psi_{a}=1$, and $\psi_{b}=0.25$.

The results above allow us also to draw a phase diagram of the system (Fig. 11). In this diagram, stable disease-free behavior (type I) is separated from epidemic behavior (type II and III) by a transcritical bifurcation, while outbreak (type II) and epidemic (type III) behaviour are separated by the heteroclinic bifurcation.

Trajectories starting in the type-II phase lead to final states in the type-I phase. In fact, the black arrow is the trajectory for $\beta =0.5,\;{\left[S_{a}\right]}_{0}=0.6$. Again, we can think of this kind of event as an inoculation, since the initial outbreak is crushed, and we are left with fewer type A susceptibles so that future outbreaks need a much higher *β* (around 1.8 in this case) to succeed.

## VIII. CONCLUSIONS

In this paper, we investigated a previously proposed model for the spreading of a disease across a network in the face of behavioral responses to the disease and intra-individual heterogeneity of epidemic parameters. To understand the dynamics of this system, we used a variety of tools, including agent-based simulation, percolation theory, moment expansions, analytical bifurcation theory, numerical integration of ODEs, and continuation.

Our results point to a phenomenon that we named *network inoculation*. Introducing a disease into a given network may lead to an outbreak that collapses and leaves the network with a different topology as agents have rewired their connections in response to the disease. Although the altered topology will be generally more heterogeneous than the initial topology, it is more resilient to disease outbreaks. In this sense, network inoculation is strongly reminiscent of immunological inoculation as in both cases contact to the pathogen leads to a response that hardens the system against future exposure to the pathogen.

Our analysis showed that the outbreak and collapse dynamics characteristics of network inoculation occur in a region bordered by two phase transitions. When viewed from a macroscopic perspective, one of these transitions is a transcritical bifurcation, whereas the other is a saddle-heteroclinic bifurcation. Network inoculation thus provides a (rare) example of a phenomenon where a global bifurcation causes a phase transition in a model that can be understood both on the micro- and macroscale.

We emphasize that network inoculation is not a peculiarity of the specific model studied here. By contrast, we expect the phenomenon to occur in a wide variety of models as soon as certain requirements are met. While the phenomenon may as well occur in other models, let us for consistency summarise the requirements of network inoculation in epidemic terms. Network inoculation can occur if there is

(1) a disease-free attractor (inoculated outcome),
(2) an endemic attractor (endemic outcome), and
(3) a variety of unstable disease-free states (initial states).

The actual inoculation strictly speaking only requires conditions 1 and 3, whereas condition 2 makes the onset of inoculation via a heteroclinic bifurcation possible.

If the first two conditions are met, there will be generally a saddle of some sort whose stable manifold marks the separatrix between the basins of the two attractors. Network inoculation will occur if the initial state is in (or on) the basin of the inoculated outcome. When parameters are changed, the separatrix will generally move, which can cause an initial state to enter or leave the basin of the inoculated outcome, in a heteroclinic bifurcation.

The conditions above require a bistability between an endemic (1) and a disease-free (2) state. While such bistability is not observed in the most simple models, it is very common in even slightly more complex models. In particular, this bistability has been observed in numerous variants of the adaptive SIS models. It therefore seems to be a robust feature of epidemiological models that appears once behavioral responses to the disease are modelled.

Furthermore, we require the existence of multiple disease-free states with different stability properties. While the simplest epidemiological models have only a single disease-free state, multiple disease-free states naturally appear as soon as an additional macroscopic variable exists.

Network inoculation was not observed in the previous investigations of the adaptive SIS models. While this model shows robust bistability, it has only a unique disease free state and hence does not meet the requirements of network inoculation. Likewise, network inoculation was not observed in the previous models of epidemics in heterogeneous populations. In these models, there are naturally multiple disease-free states which differ in the connectivity of the different classes of individuals. However, because these previous models did not consider adaptive rewiring of links, the connectivities of the different classes of agents are parameters, rather than dynamical variables. Thus, the different disease-free states are not observed simultaneously for one choice of parameters, hence again inoculation-type dynamics cannot occur.

Once intra-individual heterogeneity and adaptive network rewiring are both considered, multiple disease free states that differ in the connectivity of classes of individuals occur robustly. Because adaptive rewiring can change these connectivities, they are now dynamical variables, and the multiple disease-free states can be observed simultaneously, for a given set of the remaining parameters. When multiple disease-free states exist, the generic expectation is that they will have different stability properties at least in some region of the parameter space, and thus there will, in general, be a parameter region where the conditions for network inoculation in the narrow sense are met.

Because bistability between endemic and disease free states has proven to be a very robust feature of adaptive epidemiological models, we can moreover expect the onset of network inoculation via the heteroclinic bifurcation to be a common phenomenon. Both ingredients, the adaptive response of the network to the disease and intra-individual heterogeneity, are known to exist in the real world. In the light of the arguments above, we expect network inoculation, and its onset via the heteroclinic bifurcation to occur whenever these two ingredients are combined in the same model.

Thus, it seems that the reason why network inoculation has not been observed in the past is not the phenomenon itself is rare, but rather that the models that have been studied so far have been too strongly simplified to capture this, potentially common, phenomenon.

## DATA STATEMENT

This study did not use any primary research data.

## APPENDIX: LINK DENSITY DIFFERENTIAL EQUATIONS

The additional differential equations for link densities are as follows:

$$\begin{array}{c} \frac{d[S_{b}S_{b}]}{dt}=\mu [S_{b}I_{b}]-2\beta \psi_{b}(\frac{\left[S_{b}S_{b}][S_{b}I_{a}\right]}{\left[S_{b}\right]}+\frac{\left[S_{b}S_{b}][S_{b}I_{b}\right]}{\left[S_{b}\right]})\\ +\frac{\omega [S_{b}]}{\left[S_{a}]+[S_{b}\right]}([S_{b}I_{a}]+[S_{b}I_{b}]), \end{array}$$ (A1)

$$\begin{array}{c} \frac{d[S_{a}S_{b}]}{dt}=\mu ([S_{b}I_{a}]+[S_{a}I_{b}])-\beta \psi_{a}(\frac{\left[S_{b}S_{a}][S_{a}I_{a}\right]}{\left[S_{a}\right]}+\frac{\left[S_{b}S_{a}][S_{a}I_{b}\right]}{\left[S_{a}\right]})-\beta \psi_{b}(\frac{\left[S_{a}S_{b}][S_{b}I_{a}\right]}{\left[S_{b}\right]}+\frac{\left[S_{a}S_{b}][S_{b}I_{b}\right]}{\left[S_{b}\right]})+\frac{\omega [S_{b}]}{\left[S_{a}]+[S_{b}\right]}([S_{a}I_{a}]+[S_{a}I_{b}])+\frac{\omega [S_{a}]}{\left[S_{a}]+[S_{b}\right]}([S_{b}I_{a}]+[S_{b}I_{b}]), \end{array}$$ (A2)

$$\begin{array}{c} \frac{d[S_{a}I_{a}]}{dt}=2\mu [I_{a}I_{a}]-(\mu +\beta \psi_{a}+\omega )[S_{a}I_{a}]+2\beta \psi_{a}(\frac{\left[S_{a}S_{a}][S_{a}I_{a}\right]}{\left[S_{a}\right]}+\frac{\left[S_{a}S_{a}][S_{a}I_{b}\right]}{\left[S_{a}\right]})\\ -\beta \psi_{a}(\frac{\left[S_{a}I_{a}][S_{a}I_{a}\right]}{\left[S_{a}\right]}+\frac{\left[S_{a}I_{a}][S_{a}I_{b}\right]}{\left[S_{a}\right]}), \end{array}$$ (A3)

$$\begin{array}{c} \frac{d[S_{b}I_{b}]}{dt}=2\mu [I_{b}I_{b}]-(\mu +\beta \psi_{b}+\omega )[S_{b}I_{b}]+2\beta \psi_{b}(\frac{\left[S_{b}S_{b}][S_{b}I_{a}\right]}{\left[S_{b}\right]}+\frac{\left[S_{b}S_{b}][S_{b}I_{b}\right]}{\left[S_{b}\right]})\\ -\beta \psi_{b}(\frac{\left[S_{b}I_{b}][S_{b}I_{a}\right]}{\left[S_{b}\right]}+\frac{\left[S_{b}I_{b}][S_{b}I_{b}\right]}{\left[S_{b}\right]}), \end{array}$$ (A4)

$$\begin{array}{c} \frac{d[S_{a}I_{b}]}{dt}=\mu [I_{a}I_{b}]-(\mu +\beta \psi_{a}+\omega )[S_{a}I_{b}]+\beta \psi_{b}(\frac{\left[S_{a}S_{b}][S_{b}I_{a}\right]}{\left[S_{b}\right]}+\frac{\left[S_{a}S_{b}][S_{b}I_{b}\right]}{\left[S_{b}\right]})\\ -\beta \psi_{a}(\frac{\left[S_{a}I_{b}][S_{a}I_{a}\right]}{\left[S_{a}\right]}+\frac{\left[S_{a}I_{b}][S_{a}I_{b}\right]}{\left[S_{a}\right]}), \end{array}$$ (A5)

$$\begin{array}{c} \frac{d[S_{b}I_{a}]}{dt}=\mu [I_{a}I_{b}]-(\mu +\beta \psi_{b}+\omega )[S_{b}I_{a}]+\beta \psi_{a}(\frac{\left[S_{a}S_{b}][S_{a}I_{a}\right]}{\left[S_{a}\right]}+\frac{\left[S_{a}S_{b}][S_{a}I_{b}\right]}{\left[S_{a}\right]})\\ -\beta \psi_{b}(\frac{\left[S_{b}I_{a}][S_{b}I_{a}\right]}{\left[S_{b}\right]}+\frac{\left[S_{b}I_{a}][S_{b}I_{b}\right]}{\left[S_{b}\right]}), \end{array}$$ (A6)

$$\begin{array}{c} \frac{d[I_{a}I_{a}]}{dt}=-2\mu [I_{a}I_{a}]+\beta \psi_{a}[S_{a}I_{a}]+\beta \psi_{a}(\frac{\left[S_{a}I_{a}][S_{a}I_{a}\right]}{\left[S_{a}\right]}+\frac{\left[S_{a}I_{a}][S_{a}I_{b}\right]}{\left[S_{a}\right]}), \end{array}$$ (A7)

$$\begin{array}{c} \frac{d[I_{b}I_{b}]}{dt}=-2\mu [I_{b}I_{b}]+\beta \psi_{b}[S_{b}I_{b}]+\beta \psi_{b}(\frac{\left[S_{b}I_{b}][S_{b}I_{a}\right]}{\left[S_{b}\right]}+\frac{\left[S_{b}I_{b}][S_{b}I_{b}\right]}{\left[S_{b}\right]}). \end{array}$$ (A8)
